# The Effect of Various Vehicles on the Naproxen Permeability through Rat Skin: A Mechanistic Study by DSC and FT-IR Techniques

**DOI:** 10.15171/apb.2016.03

**Published:** 2016-03-17

**Authors:** Anayatollah Salimi, Najme Hedayatipour, Eskandar Moghimipour

**Affiliations:** Nanotechnology Research Center, Department of Pharmaceutics, Ahvaz Jundishapur University of Medical Sciences, Ahvaz, Iran.

**Keywords:** Naproxen, permeability, Mean transition temperature, Thermotropic behavior, Diffusion coefficient, Flux

## Abstract

***Purpose:*** The purpose of the present investigation was to evaluate the effectiveness of different vehicles on drug permeability and microstructure of intercellular or lipids in SC layer of skin.

***Methods:*** Pre-treated skin of rat using some vehicles including Labarac PG ,Transcutol P, tween 80, span 80 and propylene glycol (PG), were mounted on specialized design franz-diffusion cell was used to assess naproxen permeation and parameters such as permeability coefficients and state flux (J_ss_) were evaluated. Any differences in peak position and also change in symmetric and asymmetric stretching of C-H bond, lipid ester carbonyl stretching in SC, C=O stretching (Amide I) and C-N stretching of keratin (Amide II) absorbance using Fourier transform infrared spectroscopy (FTIR) were considered to investigate the enhancing mechanism. DSC method was utilized to compare their mean transition temperature (Tm) and enthalpies (ΔH).

***Results:*** Steady-state flux (Jss), permeability coefficient (Kp) and diffusion coefficient (D) were significantly (p<0.05) increased by using their span80 showed the biggest enhancement ratio (ERflux) and Transcutol P, Labrafac PG, Tween 80 and Propylene glycol were at the next levels. In comprised to hydrated rat skin the maximum increase in diffusion coefficient was for Tween 80(p<0.05), Lipid fluidization, lipid disruption structure and the irreversible denaturation of proteins in the SC layer of skin by span 80, Tween 80, Labrafac PG, Transcutol P and propylene glycol, were indicated by FT-IR and DSC techniques.

***Conclusion:*** It is concluded that naproxen permeation through rat skin may be facilitated by utilizing the vehicle systems. Lipid fluidization and lipid extraction are among suggested mechanisms.

## Introduction

Nowadays, dermal delivery of drugs is considered as an important way for administration of drugs. There are many advantages with transdermal drug delivery( TDD), although only a few drugs with acceptable release profiles have used in therapy. Utilization of vehicles that affect the skin barrier function is one of the classic strategies of permeation enhancement. Some of these vehicles have well characterized actions on the stratum corneum, but the majority is still selected using empirical criteria. The skin, our body^’^s largest organ, is generally considered as an impermeable protective barrier against mechanical, chemical, microbial, and physical hazards. The success of a topical drug to be used for systemic drug delivery depends on the ability of the drug to penetrate via skin in sufficient quantities to achieve the desired effect.^1^

Avoiding first pass metabolism , sustained and controlled delivery of drugs, decrease of dose frequency, proper patient compliance and localization of drugs in target site, avoidance of the risks of injections, and reduction in toxic level of drugs are among TDD advantages.^2-6^

Skin permeation of drugs is an essential stage percutaneous absorption. Permeation is the penetration from one layer into another layer of skin. The lipid matrix of stratum corneum layer acts a meaningful role in determining the permeability of substances through the skin. The physicochemical properties of both drug and vehicle play an important role in determining percutaneous absorption.^7^

Skin permeation can be improved by the several strategies: increasing the diffusivity in the skin by disturbance of SC lipid matrix, strengthening drug solubility in the skin, enhancing the degree of saturation of the drug in formulation.^8^

The main principle of transdermal drug delivery is passive permeation of drug through skin ,that often depends the solubility and partition coefficient of the drug molecule. Two main strategies for improvement permeation of drugs are reversible enhancing effect of vehicle and modifications of drug thermodynamic property.^7^ So, selection of a suitable vehicle for the preparation of topical formulations greatly affects on the drug delivery.

Several vehicles including oleic acid, propylene glycol and water are considered as permeation enhancers working by some mechanisms including disruption of the organized intercellular lipid structure of the stratum corneum, fluidization of the stratum corneum lipids, cellular proteins alteration, and extraction of intercellular lipids by non-polar solvents.^6-8^

To develop transdermal drug delivery systems, investigation of microstructure of intercellular or lipids in SC layer of skin is required. In the recent studies, organization of lipids and skin microstructure have been examined using various techniques including differential scanning calorimetry( DSC) and Fourier transform infrared spectroscopy (FT-IR).^9^ The FTIR analysis of skin can be practical tool for studying the interaction between chemical enhancers materials with SC that provides bands at different wave numbers.^10,11^

Several infrared spectral bands of the skin are attributed to vibration of protein and lipid molecules in the SC.^12^ The lipid vibration are a good index from which to evaluate the microstructure of the lamellar lipids form in the intercellular area in the SC layer of skin .Some different spectral regions of the SC was expected to have various bands, -CH2 symmetric vibration ( near 2850 cm^-1^) and –CH2 asymmetric vibration ( near 2920cm^-1^) and amide I (about 1650cm^-1^) and amide II (near 1550cm^-1^) stretching vibrations of SC protein have been reported. An increase in wave number and width of –CH2 stretching peaks is due fluidization of the stratum corneum lipids.^13-15^ The frequencies of amide I and amide II bands, especially amide I band, are sensitive and shift to lower or higher frequencies according to the change in protein conformation. Alteration of the amide I (C=O stretching) bands shows secondary structure of keratin, which may interference with H-O-H bending vibrationof water at 1645cm^-1^. The amide bonds along with 1645cm^-1^ band are sensitive to H-bond change in the SC.^13^

To recognize the mechanism by which the properties of enhancers/retardants change in a given vehicle, molecular studies of whole rat skin was carried out using DSC and FTIR.

The rationale for use of FTIR technique to understand mechanism of penetration modifier was that the treatment with enhancers/retardants, sometimes yield a shift in specific band position to higher or lower wavenumber or lead to change in the intensity of the signal observed at that band position.

If the shift is to higher wavenumber (blue shift), it indicates SC membrane (lipid bilayer) fluidization that in change contribute to disruption of the barrier properties that probably causes the substance permeation enhancement through the SC.^16^ On the other hand, lipid groups are oriented again, a phenomenon that that causes a shift to lower wave number (e.g. red shift) and strengthening of subcutaneous barrier properties which finally slows down the entrance of permeant through the skin.^16^ If the penetration modifier performs by affecting on lipid pathway, the phase transition of the lipids is showed by increase/decrease in the band position (wavenumber) of the signals at 2920, 2850, and near1738 cm^−1^.

The quantity of proteins and lipids in SC layer is presented by area or height of the bands and any alteration in the intensity of the peaks may be due to strengthening or extraction of subcutaneous lipids. The mechanism is also suggested for the treatment with penetration enhancers. They may also increase the intensity at specific band indicating retardation when retardants are utilized.^16^

Thermal analysis methods such as differential scanning calorimetry (DSC) have been utilized to investigate thermal transitions in mammalian stratum corneum. The stratum corneum (SC) is the outermost layer of the epidermis and is primarily responsible for the skin’s barrier function.^11^

The DSC technique is widely used for characterization the melting of lipids and the phase transition of lipid bilayers and protein denaturation in SC layer. To obtain more detailed information about lipid components and protein conformational stability of the whole skin rat treated with pure vehicle, a DSC study was programmed. By comparing mean transition temperature (T_m_) and enthalpies (H), thermotropic behavior of treated skin was assessed. Any transition in T_m_ to lower degrees may be due to lipid disruption in bilayer and irreversible protein denaturation in SC. While, enthalpy decrease was related to lipid fluidization in lipid bilayers and protein – lipid complexes.^13,15,17-19^ Naproxen is a propionic acid derivative drug widely used as analgesic, antipyretic and for relief of symptoms of dysmenorrheal pain.^12^ Common adverse effect of NSAIDs including, GI ulcer, accompanied by anemia as a result of bleeding. To reduce the risk of gastric irritation and toxicity, and also to gain a more effective therapy, transdermal administration is a promising route. It also enables sustained and controlled release of drugs and increases patient compliance.^20^

The study was aimed to evaluate the effectiveness of some hydrophobic and hydrophilic vehicles on naproxen *in vitro* skin permeability of through Excised Whole Rat Skin and also the study of biophysical characteristics of Rats’ stratum corneum by FT-IR and DSC techniques in order to design a new drug delivery system for skin delivery of naproxen.

## Materials and Methods

Naproxen was obtained from Pars Darou (Iran), Propylene glycol dicaprylocapraye (Labrafac PG, Diethylene glycol monoethyl ether (Transcutol P) was donated as gift by GATTEFOSSE Company (France). Span 80, Propylene glycol( PG), and Tween 80 were purchased from Merck (Germany). All solvents and chemicals were of analytical grade. Also, Freshly double distilled water was used in the experiments

### Solubility Tests

The solubility of naproxen was determined in Transcutol p, Labrafac PG, Tween 80, Span 80 and PG. A definite quantity of naproxen was added to 5 ml of the solvent. The mixture was immersed in a water bath and equilibrated at 37 °C for 24 hrs. Then, suspension was centrifuged for 10 min at 3000 rpm, filtered, diluted and the dissolved drug was quantified spectrophotometrically at 271 nm.^21^

### Animal Studies

Male adult Wistar rats (weighing 175 - 225 g) and aged 8-10 weeks were prepared from Animals Laboratory, Jundishapur University of Medical Sciences, Ahvaz, Iran. Their abdominal skin was shaved using an electric clipper, taking care not to damage the skin. Prior to sacrificing, they were anaesthetized with ether. Abdominal full-thickness skin was removed and using cooled pure acetone solution with 4 °C any extraneous subcutaneous fats cleaned from the dorsal side. The thickness of whole skin was determined using a digital micrometer (AAOC, France). The animals were treated according to the principles for the care and use of laboratory animals and the procedures followed with standard international guidelines.^22^

### Differential scanning calorimetry (DSC) 

The solvents, which induced skin structural alteration, were determined using a DSC instrument (DSC^1^ system, Mettler Toledo). About 5-10 mg of each skin sample was placed in a hermetically sealed aluminum pan. Simultaneously, an empty sealed pan was concurrently utilized as reference. Skin samples were exposed to heat ranging from 20 to 200°C (scan rate: 5 °C/min). Enthalpy (ΔH) values were determined from endothermic and exothermic transitions of the thermo grams.^2^

### FT-IR spectroscopy

Skin samples were treated by Labrafac PG, Transcutol P, Span 80, Tween 80, PG for 24 hrs,then vacuum-dried (650 mm Hg, 25 ± 1 °C) for 1 h and finally stored in desiccators to remove traces of solvent . The skin samples were analyzed in 4000 to 500 cm^-1^ scan range using an FT-IR facility ( Uker, Vertex70, Germany).^2^

### Permeability Experiments

Specially designed vertical diffusion cells (with an effective diffusion area of approximately 4.9 cm^2^) were utilized to evaluate in vitro naproxen permeation. The receptor compartment was filled with 25ml phosphate buffer solution (PBS, pH 7.4). Whole skin sample, hydrated prior to use, was mounted between the donor and receptor compartments of the cell with the stratum corneum facing the donor medium. 1 % (w/v) solution of naproxen in each solvent was placed in donor compartment. The diffusion cells were placed on a heater-stirrer ( 37 ± 0.5 °C) and the receptor phase was stirred continuously at 200 rpm using small magnetic bars . At each interval time (0.5, 1, 2, 3, ……, 56 h), a 2ml sample was withdrawn from the receptor phase, and replaced by an equivalent volume of PBS to maintain sink condition. A UV spectrophotometer was utilized to detect the permeated amount of naproxen in derived samples.^2,22^

### Data analysis and statistics

Cumulative permeated naproxen per unit area was calculated by dividing the corrected naproxen concentration by the area of the exposed area of the skin to donor solution. Steady-state flux (mg/cm^2^/h) was calculated from the linear portion of the slope of the permeation curve. Permeability coefficient(Kp, cm/h) through the skin for naproxen was calculated as in Eq1.^23,24^: naproxen concentration was corrected for sampling effects according to Eq1.^23,24^

Kp = J_ss_/C_v_. …………….…………….. (1)

where is Jss and Cv were steady-state flux and naproxen concentration in donor phase,respectively.

All of the experiments were carried out in triplicateand the results were presented as mean±SE, using one-way analysis of variance (ANOVA), the data were statiscally analyzed and p values less than 0.05 were considered as significant differences.

## Results and Discussion

### Solubility Study

Table 1 shows the solubilities of naproxen in different vehicles. Its solubilities in Span 80, Propylene glycol, Transcutol P, Labrafac PG, Tween 80 and water was 15.033 mg/ml, 14.12mg/ml,13.87 mg/ml, 13.697mg/ml, 0.567mg/ml and 0.036 mg/ml, respectively .

**Table 1:** Solubility of naproxen in various solvents at 37°C (mean±SD,n=3)

**vehicle**	**Solubility(mg/ml)**
Span 80	15.033±0.208
Propylene glycol	14.120±0.368
Transcutol p	13.87±0.023
Labrafac PG	13.697±0.536
Tween 80	0.567±0.012
Water	0.036±0.002

### FT-IR Spectroscopy

**Figure 1:**
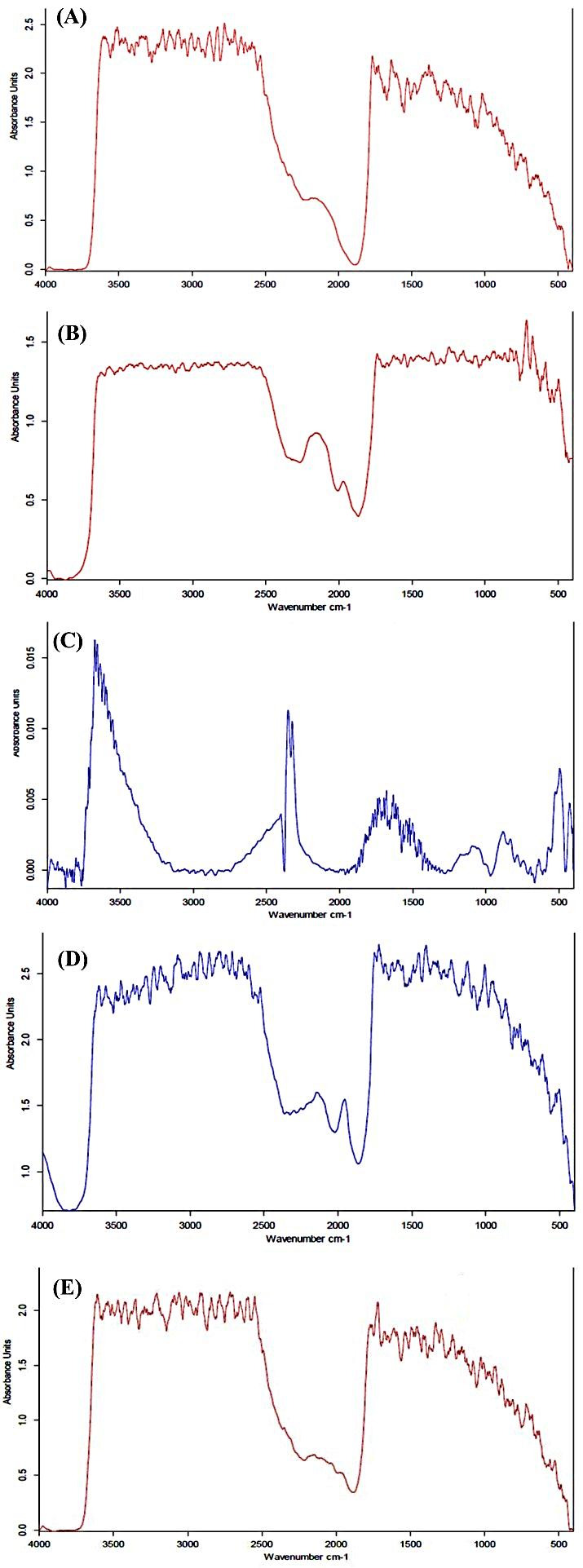
FT-IR spectra of whole skin abdominal rat after treatment with (A): Propylene glycol, (B): Labrafac PG, (C): Span 80, (D): Transcutol p, (E): Tween 80.

Figure 1, Table 2, Table 3 and Table 4 show spectral analysis of samples, regarding any change in position of peaks and also their intensities from intensities from 4000cm^-1^-500cm^-1^.

Any O-H and N-H stretches from protein, water and lipid are observed between 3000-3600cm^-1^, while symmetric and asymmetric stretching bonds of terminal methyl groups of dermal lipids are presented around 2948.02 and 2821.65 cm^-1^. The lipid ester carbonyl stretching in SC showed at 1723.62 cm^-1^ position. Also, the stretching of lipid ester in stratum corneum and amide I (C=O stretching) and amide II (C-N stretching) linkage of the helical secondary structure found in epidermal keratin are presented at 1652.78 and 1572.27 cm^-1^.^15-17^

The spectra of PG treated rat skin show changes in peak height and wave numbers. The lack of significant correlation between drug solubility in PG and ER_flux_ probably indicates that the high hydrogen bonding capacity of PG decreased affinity of skin for bonding with naproxen during treatment with this vehicle. Hence, the effect of vehicles on drug solubility in stratum corneum and hydrogen bonding formation between skin and drug are important factors for drug partitioning. ER_D_ results after skin treatment with PG correlate with FT-IR and DSC observations so that propylene glycol interacts mainly with proteins in stratum corneum layer, Red shift was observed in PG-treated rat skin at wave number (2929.5cm^-1^, indicating reorientation within the lipid groups that causes strengthening of SC barrier properties, thus resulting in decrease in naproxen transport across the skin. Relatively red shift in 1722.7cm^-1^ band was observed in PG treated skin rat ,indicating formation of strong hydrogen bonds within the lipid structures. Skin treatment with permeation modifiers might have led to lipid extraction from SC layer, or might have increased the intensity at the particular band indicating retardation in the case of retardants.^13^

IR spectra of Tween 80 treated rat skin represented blue shifts and significant decreases in height of peaks height in the 3010.75, 2840.74, 1770.18, 1653, 1591.73 cm^-1^ wave numbers.

Red shifts was observed in labrafac PG-treated rat skin and height of peaks at wave numbers (2895.27, 2778, 1716.94 cm^-1^) decreased significantly, and blue shift in which peak height of the wave numbers (1669.4 and 1591.82cm^-1^) decreased significantly. Relatively red shift in band 1716.94 cm^-1^ was observed in labrafac PG treated skin rat, indicating formation strong hydro genic bonds within the lipid molecules that causes strengthening of SC barrier properties, thus resulting in decrease in diffusion coefficient than tween 80. Height of peaks of wave numbers (2860.64, 1764.86, 1694.84, 1599.9 cm^-1^) were significantly decreased when the skin was treated with span 80 and blue shifts at 2860.64, 1764.86, 1694.84, 1599.9 cm^-1^and disappeared at 29486.02cm^-1^. The spectra for rat skin treated with transcutol P indicate red shifts with lowering of in peak height of the wave number (2836.97cm^-1^) and blue shift in which height of peak numbers (2895.24, 1729.69 and 1579.46 cm^-1^) decrease.

Lipid fluidization, lipid disruption and the irreversible denaturation of proteins in the SC layer of skin by tween 80, span 80 and labrafac PG as indicated by FT-IR and DSC, is the main factor for higher ER_flux_ and ER_D_ ratios in comparison with control.

### Differential Scanning Calorimetery (DSC)

**Figure 2:**
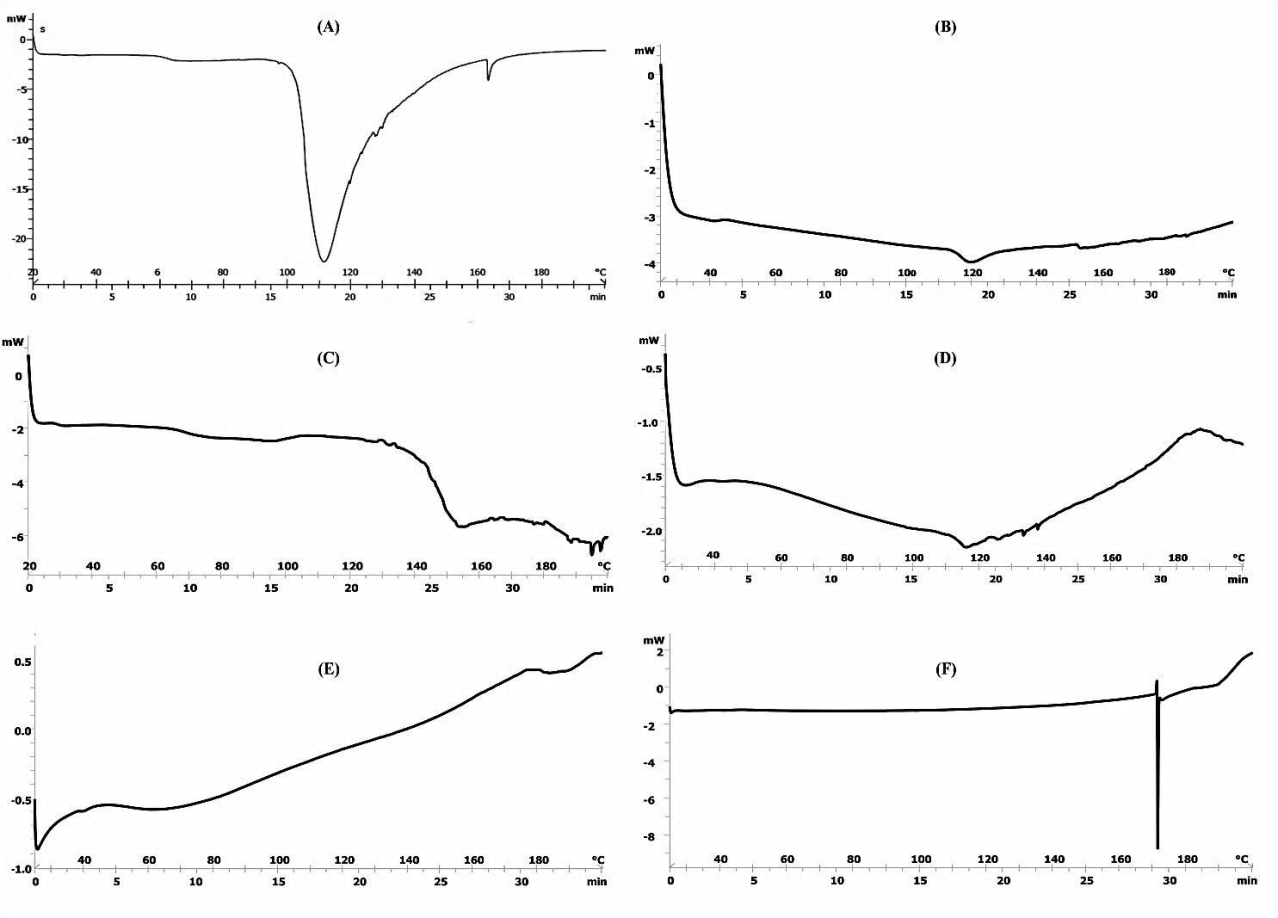
DSC Thermograms of whole skin abdominal rat after treatment with (A) Water (B) Labrafac PG (C) Propylene glycol (D) Span 80 (E) Transcutol P (F) Tween 80

Mean transition temperature (T_m_) and their enthalpies (ΔH) were used to evaluate thermotropic behavior of treated skin. Table 5 shows transition temperatures and enthalpies while Figure 2 shows thermograms. Tm_1_ and Tm_2_ resulted from hydrated rat skin were 67.5 and 112°C, respectively, indicated melting of lipids and intracellular keratin irreversible denaturation.^2^ Vaddi et al were obtained three endothermic transition at 62, 79, and 95°C in the thermotropic behavior of rat skin^21^ while Shakeel et al.^24^ observed four endothermic transitions at 34, 82, 105, and 114°C. Kaushik et al reported in human dermal DSC graphs and observed three endothermic transition peaks at temperatures 59-63°C (Tm_1_), 75-82°C (Tm_2_) and 99.5-120°C (Tm_3_).^16^ They suggested that Tm_1_ was due to transformation of lipid forms from a lamellar to disordered state, Tm_2_ corresponds to protein-lipid complex melting point,^16^ or the disruption of polar head groups of lipids^14-16^ and Tm_3_ is known to occur during the proteins irreversible denaturation,^14^ respectively. It was observed that span 80, labrafac PG to lower melting points and ΔH_1_ decreased in comparison with hydrated rat skin, shifted Tm1. In addition, Tm_1_ and Tm_2_ were disappeared by tween 80, while Tm_1_ was shifted by PG and transcutol P to higher melting point and ΔH_1_ increased in transcutol P when compared with control. Also, all the vehicles shifted Tm_2_ indicating increase in melting point (with the exception of Tween 80 and Transcutol P) and decrease enthalpies that may be due to protein denaturation.

**Table 2:** Decrease in mean peak height (± SD), compared with control (untreated skin) of asymmetric (Asy) and symmetric (Sym) C-H stretching and C=O stretching absorbance of abdominal hydrated whole skin rat following treatment with different vehicles (mean ± SD, n = 3)

**vehicles**	**Asymmetric C-H stretching**		**Symmetric C-H stretching**		**C=O stretching of lipid ester**	
	**Peak height**	**%D**	**Peak height**	**%D**	**Peak height**	**%D**
Control	4.877±0.0015	-	5.026+0.0026	-	4.999±0.0065	-
PG	0.021±0.002	99.56±1.05	0.023+0.0041	99.54+1.547	0.013±0.002	99.73±1.41
Span 80	0	100	1.95+0.041	61.2±1.004	1.874±0. 018	62.5±1.015
Tween 80	2.129±0.066	56.34±1.84	2.115±0.055	57.91+1.804	1.872±0.025	62.55±1.215
Labrafac PG	2.092 ±0.008	57.1 ±1.93	1.999±0.078	59.01±2.11	1.944±0.045	61.1±1.312
Transcutol P	1.888±0.012	61.28±2.01	1.850±0.114	63.19±2.29	1.640±0.051	67.19±1.493

*%Decrease in peak height(%D) =( peak height from untreated whole skin - peak height from solvent treated whole skin)/ peak height from untreated whole skin x 100

**Table 3:** Decrease in mean peak height (± SD), compared with control (untreated skin) of C=O stretching (Amide I)and C-N stretching of keratin(Amide II )absorbance of abdominal hydrated whole skin rat following treatment with different vehicles (mean ± SD, n = 3)

**vehicles**	**C=O stretching of keratin**		**C-N stretching of keratin**	
	**Peak height**	**%D**	**Peak height**	**%D**
Control	4.952±0.021	-	4.840+0.045	-
PG	0.012±0.002	99.75±1.211	0.007±0.0003	99.85±2.18
Span 80	1.725±0.089	65.16±1.15	1.953±0.053	59.64±1.71
Tween 80	1.774±0.011	64.17±2.05	1.806±0.007	62.68±1.92
Labrafac PG	1.806±0.026	63.52±1.88	1.857±0.013	61.63±1.58
Transcutol P	1.555±0.064	68.598±2.014	1.719±0.037	64.48±1.71

**Table 4:** FT-IR Peak wave numbers(cm^-1^) changes compared with control (untreated skin) and abdominal hydrated whole skin rat following treatment with different vehicles. (mean ± SD, n = 3)

**vehicles**	**C-H stretching Asy**	**C-H stretching Sym**	**C=O stretching of lipid ester**	**Amide I**	**Amide II**
Control	2948.02±0.115	2821.65±0.42	1723.65±0.55	1652.78±0.35	1572.27±0.22
PG	2952.9±0.6	2754.62±0.53	1724.34±0.45	1677.75±0.47	1539.97±0.45
Span 80	Deleted	2860.64±22	1764.86±0.42	1694.84±0.38	1599.9±0.62
Tween 80	3010.75±1.02	2840.74±0.27	1770.18±0.19	1653±0.9	1591.73±0.58
Labrafac PG	2895.27±0.62	2778±1.2	1716.94±0.62	1669.4±0.56	1591.82±0.72
Transcutol P	2936.97±0.35	2895.24±1.4	1729.69±0.48	1652.58±0.81	1579.46±0.63

**Table 5:** Effect of vehicles on the thermal properties of hydrated whole abdominal rat skin (mean ± SD, n = 3)

**Treatment**	**Transition temperature (°C)**		**Enthalpy (mj/mg)**	
	***Tm***_1_	***Tm***_2_	***ΔH***_1_	***ΔH***_2_
Hydrated control	67.5±2.1	112±6.6	7.01±0.4	552.1±19.5
PG	77.5±4.2	132±5.9	6.05±0.6	16.4± 2.2
Span 80	38±0.53	117±2.1	0.051±0.006	0.711±0.012
Tween 80	deleted	deleted	0	0
Labrafac PG	44.6±0.63	120±2.6	0.103±0.075	2.573±1.25
Transcutol P	68±2.12	deleted	8.925±2.658	-

Tm_1_ = mean transison temperature of lipids; SCTm_2_= mean transition temperature of irreversible denaturation of intracellularstratum corneum keratin; ΔH_1_ = transition enthalpy of lipid phase SCΔH_2_ = transition enthalpy of keratin phase SC

### In vitro permeability experiments 

**Figure 3:**
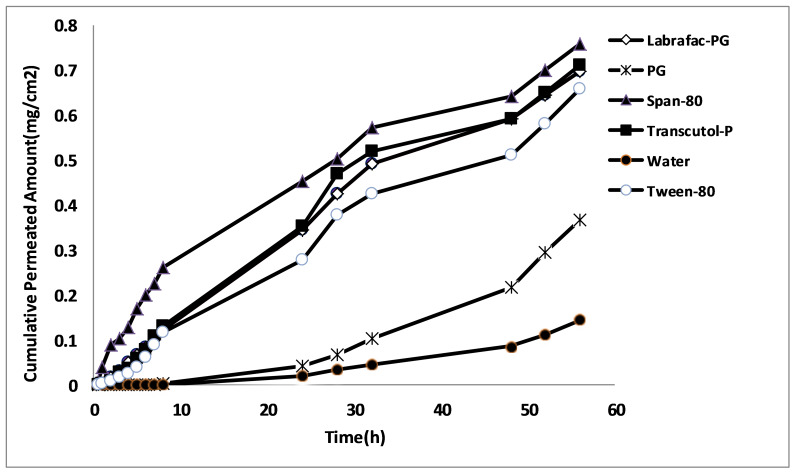
The amount of naproxen permeated from various vehicles across the rat abdominal skin

The effect of vehicles on naproxen permeability (hydrated skin as control) is expressed in Table 6 as ER_flux_, was considered to express the impact of different vehicles on the drug permeability. Hydrated rat skin was used as blank, ER_D_ (drug diffusion coefficient after and before skin pretreatment with vehicle) and ER_p_ (ratio of drug permeability coefficient after and before skin pretreatment with vehicle). Figure 3 is shown the amount of naproxen permeated from various vehicles across the rat abdominal skin. The results indicated that in comparison to control, naproxen permeation through rat skin was increased in presence of all of vehicles. The drug diffusion coefficients are significantly increased by solvents, with the highest value of ER_D_ for Tween 80 and followed by Transcotul P, labrafac PG, PG, and Span 80, indicating that their flux was more significantly increased than diffusion coefficient values.

**Table 6:** Effect of vehicles on derived permeation parameters for naproxen (mean ± SD, n = 3)

**Solvent**	**ER**_flux_	**ER**_D_	**ER**_P_
Span 80	5.364±0.014	1.150±0.004	5.408±0.117
Transcutol P	4.455±0.071	3.116±0.070	4.476±0.053
Labrafac PG	4.424±0.081	2.021±0.073	4.443±0.139
Tween 80	3.970±0.147	16.176±1.267	3.975±0.091
PG	2.909±0.180	1.228±0.138	2.913±0.011

ER_flux_ = ratio of flux after and before treatment with vehicle; ER_D_ = ratio of diffusion coefficient after and before treatment with vehicle ER_P_= ratio of permeabilitycoefficient after and before treatment with vehicle

All of the vehicles significantly increased permeability coefficient compared with water solution of naproxen (*p*<0.05). It can be deduced that the applied vehicles with different lipophilic characteristics increased naproxen solubility in stratum corneum and increased partitioning, flux and permeability. The best enhancing effect was observed by Span 80, followed by Transcutol P, Labrafac PG, Tween 80, and propylene glycol. All of the vehicles significantly increased the diffusion coefficient (p<0.05). The best ER_D_ was observed by tween 80 followed by Transcutol P, Labrafac PG, propylene glycol and span 80. ER_P_ for all the vehicles was equal increasing ER_flux_. Span 80provide the best effect on naproxen flux and permeability and tween 80 provide the best effect on diffusion coefficient.

Naproxen belongs to Biopharmaceutical Classification System (BCS) class II so that the low solubility of naproxen in water suggests that the rate-limiting phase is partitioning from stratum corneum to vital epidermis so, the vehicles having various lipophilicities increased partitioning and increased flux from stratum corneum to vital epidermis. It is indicated that there is the drug solubilities in different solvents and ER_D_ (9.7-1.07) are significantly correlated, so that any decrease in drug solubility is led to ER_D_ increase. The results of this study indicates significant correlation between drug solubility in various solvents and ER_D_ (ER_D_ = 16.8-1.5 drug solubility amount in solvents, p=0.001) so that with decrease of drug solubility in solvent, ER_D_ value is increased. The low solubility of drug in solvents might have led to higher thermodynamic activity and hence less resistance of skin, thus resulting increase in diffusion coefficient.

## Conclusion

The results obtained indicate that the used vehicles increased the drug transport through rat skin. Different mechanisms including Lipid fluidization , disruption lipid structure, lipid extraction and also irreversible protein denaturation in stratum corneum by span 80, Tween 80, Labrafac PG, Transcutol P and propylene glycol are the main probable mechanisms for higher ER_flux_ and ER_D_ ratios compared to control.

## Acknowledgments

This paper is derived from the pharm D thesis of one of the authors (najme hedayati pour). Ahvaz Jundishapur University of Medical Sciences is acknowledged for providing financial support.

## Ethical Issues

Not applicable.

## Conflict of Interest

The authors report no conflicts of interest.
